# Avian and Mammalian Diversity and Abundance in Jhalana Reserve Forest, Jaipur, India

**DOI:** 10.3390/ani14202939

**Published:** 2024-10-11

**Authors:** Swapnil Kumbhojkar, Anil Mahabal, Shrey Rakholia, Reuven Yosef

**Affiliations:** 1 Jhalana Wildlife Research Foundation, Gharkul Society, Ganeshmala, Sinhagad Road, Pune 411030, India; swapnil.kumbhojkar@gmail.com; 2 Zoological Survey of India, Pune 411044, India; mahabal.anil@gmail.com; 3 The Geographic Information System (TGIS) Laboratory, Sarkari Vasahat Road, Vastrapur, Ahmedabad 380052, India; rakholias@gmail.com; 4 Eilat Campus, Ben Gurion University of the Negev, P.O. Box 272, Eilat 88106, Israel

**Keywords:** diversity, avian, mammalian, species abundance

## Abstract

**Simple Summary:**

The Jhalana Reserve Forest (JRF) is located in the heart of Jaipur, the capital of Rajasthan in India. The JRF was declared a reserve forest in 2017 by the Rajasthan Forest Department. However, the authorities needed baseline data to identify and understand the biodiversity in the JRF. It is paramount to have this data collated to understand the ecosystem and changes that occur in subsequent years. The human population from the fringe villages was observed to intrude into the reserve. The authorities constructed a wall of 3 m height to curtail this intrusion. We studied and recorded the mammal and bird species in the JRF over 2 years, 2017–2018 and 2018–2019. In the first of its studies in the JRF, camera traps were placed at 17 artificial waterholes. We recorded 39 species (bird species—18, mammal species—14, domestic species—6, and human activity). The Indian Leopard is the apex predator in the JRF, and we identified 25 individuals during our study period. The human and domestic animal activity was reduced considerably after the wall was built. Studies involving the documentation of species are critical for the authorities to implement conservation strategies.

**Abstract:**

We utilized camera traps to evaluate animal diversity, relative abundance, and the extent of anthropogenic activities in the Jhalana Reserve Forest (JRF), located in Jaipur, with a population of 3.9 million people. Between November 2017 and November 2019, camera traps were strategically deployed in the tourist zone and peripheral areas, capturing 16,328 photos. This study represents the first comprehensive baseline assessment of animal diversity in the JRF, documenting 39 species, including 18 bird species, 14 mammals, and 6 domestic species, alongside human activity. Among the 14 mammal species, 7 were carnivores. Notably, we identified 25 individual Indian leopards (*Panthera pardus fusca*) during 2017–2018, comprising 8 males and 17 females, highlighting the leopard as the apex predator in the JRF. Concurrently, domestic animals accompanied by humans were observed within the JRF. However, rigorous conservation efforts and patrols by the Rajasthan Forest Department resulted in a notable decline in human activity, from 28.04% in 2017–2018 to 3.92% in 2018–2019, with domestic animal activity reaching zero in the latter period. Consequently, the relative abundance of wildlife species increased during the study period of 2018–2019, underscoring the positive impact of conservation strategies implemented by authorities. Our findings establish that camera-trapping methodology collates definitive baseline data, assesses mammal diversity, and evaluates relative abundance in reserve forests within human-dominated landscapes. We strongly recommend a further study to assess the avifauna diversity. This study provides critical insights to inform the development and implementation of conservation strategies in similar protected areas.

## 1. Introduction

The study of biodiversity and ecological dynamics holds paramount importance in understanding and preserving our natural world. In light of this, documenting species richness and species abundance becomes a critical tool for ecological research and conservation efforts. With the advent of technology, camera traps have emerged as an invaluable asset in this realm, offering insights into the lives of elusive and nocturnal wildlife that would otherwise remain hidden from our eyes [1]. Species diversity, distribution, and population abundance are strongly influenced by changes in habitat and its use over time [2,3]. The effective planning of biodiversity monitoring and related management strategies, as well as understanding wildlife demographics, are critical for successful conservation outcomes [4]. This is particularly important in cases of island biogeography, such as the Jhalana Reserve Forest (JRF), where humans and wildlife compete for natural resources. The JRF represents a unique ecosystem that has yet to be thoroughly explored and understood. Through our studies, we collated data using systematic camera trapping, the first of its kind in the JRF. The significance of this study lies in its potential to uncover unprecedented data regarding species distribution, human intrusion patterns, and the prey base for apex predators such as leopards within this relatively new reserve. Our comprehensive two-year survey employing camera traps ensures the reliability and depth of our findings. This research aims to illuminate the intricate tapestry of life that thrives in the shadows of the JRF, offering critical insights that could guide future conservation strategies and enhance our understanding of species dynamics in newly established protected areas.

The deployment of camera traps across various ecosystems has not just advanced but revolutionized the field of wildlife research and conservation. It has opened up a whole new world of insights into the behaviors, distributions, and populations of myriad species, often with a focus on elusive and nocturnal animals. This methodological advancement is well documented in the literature, with studies highlighting its efficacy in recording species occurrences—a cornerstone of biodiversity surveys essential for determining species distribution and informing the International Union for Conservation of Nature (IUCN) status [5]. Furthermore, photographic capture-recapture techniques facilitated by camera traps have become a gold standard in estimating the abundance and density of several secretive large carnivores, including leopards [6,7,8], striped hyenas (*Hyaena hyaena*) [9,10,11,12], tigers (*Panthera tigris*) [8,13,14], snow leopards (*P. uncia*) [15], and jaguars (*P. onca*) [16]. Beyond their application in the study of mammalian fauna [17,18,19], camera traps have also proven effective in documenting avifauna within various habitats, including forests and grasslands [20,21].

Despite camera traps’ widespread application and success in diverse environments and for various species, a noticeable gap persists in the baseline data available for certain areas, notably the JRF. Established in 2017, Jhalana has remained relatively unexplored from a scientific standpoint, lacking comprehensive data that could inform conservation efforts and enhance our understanding of its ecological dynamics [22]. This absence of baseline data represents a critical knowledge gap, underscoring the necessity of this study to employ camera traps within the reserve. By doing so, we aim to not only contribute to filling this gap but also to provide a foundation upon which future research and conservation strategies can be built, tailored to the unique environmental and biological context of the JRF.

The JRF is located in the heart of Jaipur city in north-western India and is a northern, tropical, dry deciduous forest type. Apart from a limited number of species-specific studies on spiders [23], gray langurs (*Presbitys entellus* Dufresne, 1797) [24], vegetation [25], forest ecosystem services [26], and previous studies on the local population of Indian leopards (*Panthera pardus fusca* Meyer, 1794) [22,27,28,29,30,31,32], no further studies have been conducted to document the species richness of the JRF. Leopards have existed for centuries in this small island habitat, now surrounded by 3.9 million people in Jaipur’s urban areas. However, there is no systematic documentation of the diversity of birds and mammals and the associated abundance in the JRF. The paucity of data on the ecology and wildlife of the JRF prompted us to conduct a baseline study on local wildlife diversity. 

After studying the geography and climate conditions, we discovered no perennial streams, wells, or other natural water sources in the JRF. The only water sources available are artificial waterholes created by the Rajasthan Forest Department and the local nature lovers, villagers, and tourists. Wildlife has been observed to be water-dependent and concentrate at these artificial waterholes. We set up camera traps at all of the waterholes and in outlying areas. It is known that the frequency of revisiting specific camera stations is a consequence of site fidelity [33].

At the heart of our investigation into the ecological dynamics of the JRF lies a series of focused research questions aimed at elucidating the biodiversity and anthropogenic impacts within this relatively unexplored sanctuary. Our primary research objectives are to assess the diversity of animal species and their relative abundance within the reserve and to investigate the extent and nature of human activities impacting this ecosystem. Complementing these objectives is an evaluation of the efficacy of the camera trap methodology in capturing comprehensive photographic records of the species residing in the JRF. Camera traps deployed at strategic locations (waterholes) provide a robust framework throughout the reserve for generating essential baseline data.

Our hypothesis posits that the camera trap method is an effective tool for cataloging animal diversity and their relative abundance and recording human intrusion in reserve forests situated within human-dominated landscapes. We anticipate that the insights gleaned from our study will furnish the forest department with valuable data to inform and refine strategies aimed at rewilding, curtailing human intrusion, and bolstering the prey base for the leopards of Jhalana.

To address our research aims, we employed a rigorous methodological framework leveraging commercially manufactured camera traps, strategically deployed across the reserve from November 2017 to November 2019. This approach was tailored to the specific ecological characteristics and challenges of the semi-arid region of Northwest India where the JRF is located. Notably, the reserve’s lack of perennial water sources, which concentrates species activity around artificial waterholes, guided our stratified deployment of cameras at these critical sites to maximize record yield, ensuring a thorough and comprehensive study.

Our approach is underscored by its comprehensiveness-representing the first exhaustive study of its kind within the JRF, thereby providing a necessary baseline for future management and conservation efforts. By covering all locations within the reserve and compiling data without disturbing the wildlife, our study stands as a pioneering effort in conserving and understanding this unique ecosystem. Furthermore, our methodological choices—informed by the proven efficacy of camera traps in various studies and tailored to the specific context of the JRF—address the baseline data gap.

In aligning our study with these objectives, we anticipate providing foundational insights that will significantly contribute to the conservation and management strategies for the JRF. Drawing on our extensive, novel dataset, we aim to elucidate not only the patterns of wildlife diversity and distribution but also the intricate interplay between these natural inhabitants and human activities within the reserve. This research has the potential to inspire and guide effective conservation strategies, instilling a sense of hope for the future of the JRF.

## 2. Methods

### 2.1. Study Area

**Figure 1:**
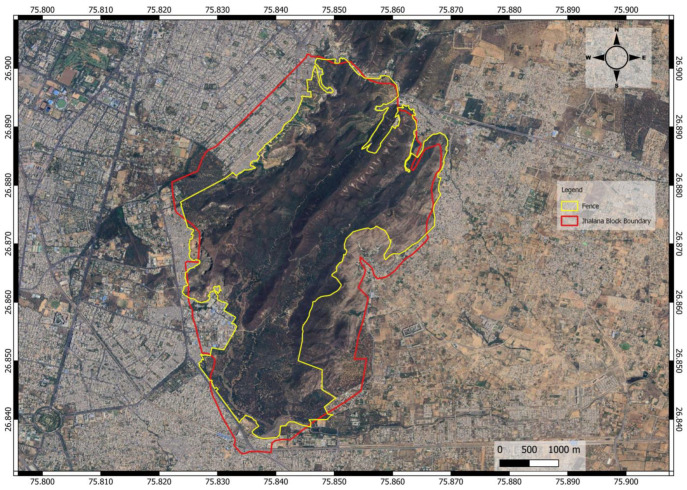
The location of the Jhalana Reserve Forest in Rajasthan State, northwestern India. The red line marks the official boundaries of the Jhalana Reserve Forest, and the yellow line marks the fence around the forest areas.

The study was conducted from November 2017 to November 2019 at Jhalana Reserve Forest (JRF), located between 26°50′13″ N, 75°50′13″ E at the southern tip and 26°54′05″ N, 75°51′03″ E in the north; and 516 m above sea level (ASL), in the southeast corner of Jaipur city, India (Figure 1).

The JRF was declared a reserve forest in 1961 under the Rajasthan Forest Act of 1953, encompassing a total area of 29 km^2^. In 2017, it was designated as a leopard reserve. During the 1980s, *Acacia tortilis* and *A. senegal* were planted in the central valley. Most ephemeral streams flow south-westerly, while higher elevations in the north form low, flat hills. Elevation in the plains ranges from 280 m in the south to 530 m in the northeast. The JRF lacks defined buffer or core areas, and a 2-m-high wall with a 3-m-high fence separates the forest area from surrounding neighborhoods and villages.

A semi-arid tropical dry deciduous forest characterizes the JRF. Tourist access is permitted through Jeep safaris on three designated routes. Due to the continuous interface between the forest and human habitats and its recent designation as a forest reserve, human encroachments into the reserve are common, as are wildlife incursions into adjacent villages and urban areas.

### 2.2. Field Methods

**Figure 2:**
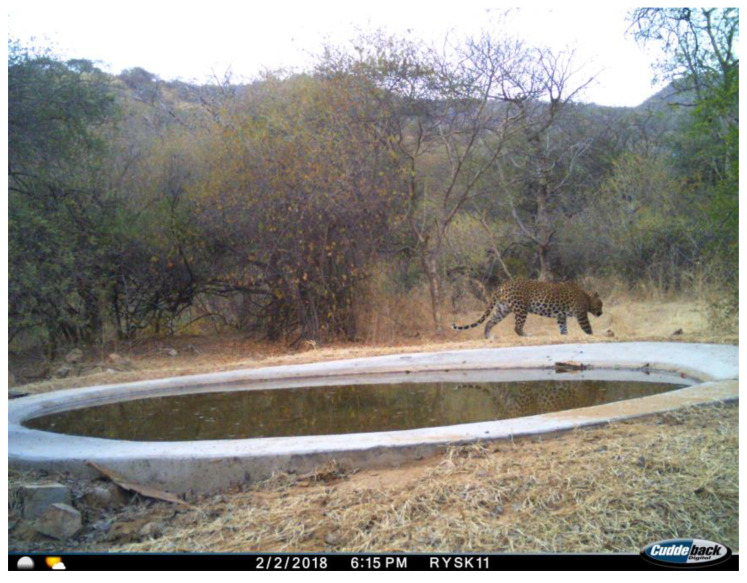
An example of documentation with camera traps of an Indian Leopard (*Panthera pardus fusca*) at a waterhole in the Jhalana Reserve Forest (JRF), Jaipur, India.

We utilized commercially manufactured Cuddeback cameras, De Pere, WI, USA (X-Change Color Model 1279) with motion sensors. No bait or lure was used to attract wildlife at any site [34]. The cameras were set with a minimum delay of 15 s between captures. Each recording period lasted two weeks, and each camera was assigned a unique identification number. A total of 21 cameras were deployed, all of which recorded the date and time of each photo (Figure 2). Overexposed images with distorted perspectives and lack of clarity were discarded (*n* = 89, 0.55%) and not included in our analyses.

Without perennial water sources in the JRF, the wildlife congregates at the 17 artificial waterholes created by the Rajasthan Forest Department. A camera was deployed at each waterhole optimally stratified to cover all waterholes in both years of the study. These ensured we captured photos of all terrestrial mammals but only some bird species. Cameras within the reserve were left untouched during the capture cycles, while the other four cameras on the periphery were removed during the day and reinstalled at night to prevent theft or damage. Cameras were installed 45 to 50 cm above the ground to cover access routes to the waterholes. In the outlying areas, camera traps were enclosed in boxes securely attached to iron bars to ensure safety. The trap locations were mapped using a GPS device.

We recorded the number of species (mean ± SD), their abundance, distribution, and human activities. Individual leopards were identified based on facial markings to estimate their abundance [22]. Of the 39 species recorded in the study area, 10 were classified as gregarious (two or more individuals in one photo, Table 1), and the remaining 29 were classified as solitary. Since photographs of gregarious species may inflate estimates of their abundance and distribution [35], we recorded one data point per photograph when more than one individual of the same species was observed.

The study was conducted over two consecutive years to understand the impact of designating the area as a reserve forest.

### 2.3. Statistical Analysis

Field data points collected from camera traps were used to compute the abundance and Shannon Diversity Index at the geolocations of the camera traps. Kruskal-Wallis, a non-parametric statistical test, was used to compute *p*-values in R library dplyr to identify statistically significant differences between the study years for diversity and abundance values. It was used to carry out year-wise comparisons for abundance and Shannon diversity values randomly sampled, derived from camera trap recordings, and further processed and mapped using the Inverse Distance Weighting (IDW) technique.

**Figure 3:**
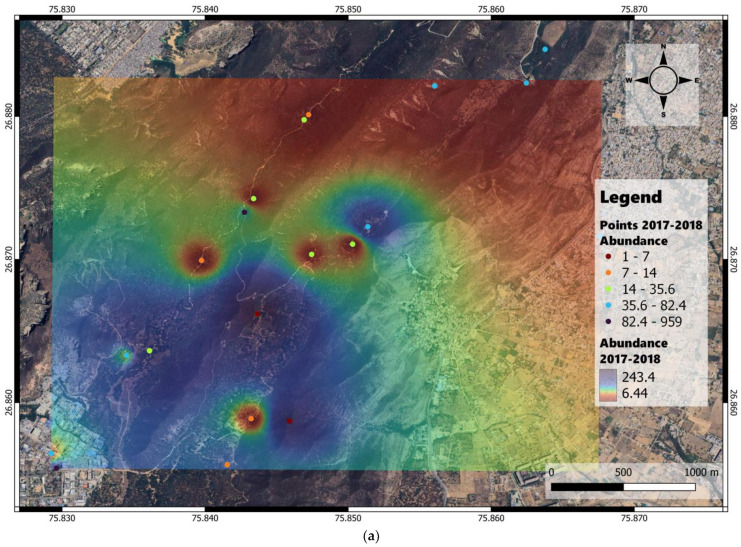

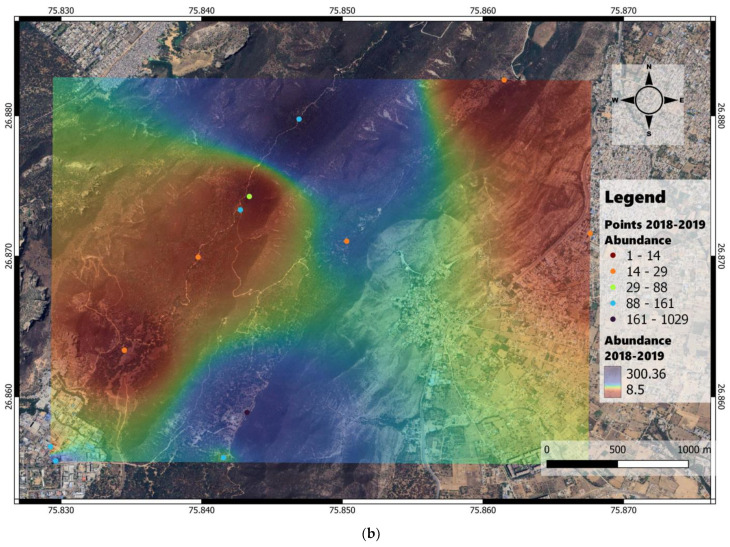
Abundance density heat maps derived through inverse distance weighting interpolation applied to recordings of camera traps deployed at point locations shown on the map of Jhalana Reserve Forest, Jaipur, India. (**a**) Abundance density map for 2017–2018 along with the camera trap observation points. (**b**). Abundance density map for 2018–2019 along with the camera trap observation points.

The geolocations varied annually based on changes in camera trap locations and waterholes. The abundance and Shannon diversity heat maps were derived through IDW interpolation based on the data obtained from camera trap recordings at point locations shown in the map (Figure 3).

Statistical comparisons were performed using R version 4.3.3. Random points (*n* > 100) were collected annually across the visible study area for this analysis. Additional mapping processes were conducted using the QGIS 3.34 spatial environment.

## 3. Results

Over the study period from 2017 to 2019, a total of 16,328 photos were captured across 23,208 trap hours, averaging 0.7 (±0.31 SD) images per hour. We recorded 39 species, including 18 bird species, 14 mammal species, 6 domestic animal species, and humans (Table 1). The relative abundance (±SD) for each species was derived from the number of photographic records. There were notable differences in taxonomic groups between the two study years: birds accounted for 25.15% and 68.33% of observations, respectively; mammals for 42.53% and 27.75%; domestic animals were only documented in 2017–2018, comprising 4.28% of observations; and humans were documented 28.04% in 2017–2018 and 3.92% in 2018–2019.

The Indian peafowl (*Pavo cristatus*) was the most frequently observed species, accounting for 24.62% of sightings in 2017–2018 and 39.94% in 2018–2019, followed by nilgai (*Boselaphus tragocamelus*) at 27.29% and 15.73%, humans (*Homo sapiens*) at 28.04% and 3.92%, and Indian leopards (*Panthera pardus fusca*) at 3.82% and 4.76%.

Among the 14 mammal species, we observed 7 carnivores, 4 artiodactyls, 1 rodent, 1 hedgehog, and 1 primate. Some species were not recorded and are present in the reserve due to low numbers or their absence in the areas where we deployed cameras. Eighteen bird species were photographed at waterholes, with jungle babblers (*Turdoides striata*), rufous treepies (*Dendrocitta vagabunda*), red-vented bulbuls (*Pycnonotus cafer*), red-wattled lapwings (*Vanellus indicus*), and blue rock pigeons (*Columba livia*) being some of the notable observations. The raptors recorded were shikras (*Accipiter badius*), Indian eagle owls (*Bubo bengalensis*), and long-legged buzzards (*Buteo rufinus*).

Among wild carnivores, we observed the Indian leopard, striped hyena, Bengal fox (*Vulpes bengalensis*), golden jackal (*Canis aureus*), small Indian civet (*Viverricula indica*), and Indian mongoose (*Herpestes edwardsii*). Herbivores frequently recorded included nilgai, chital (*Axis axis*), sambar deer (*Rusa unicolor*), and black-naped hares (*Lepus nigricollis*). Other vertebrates, such as the crested porcupine (*Hystrix indica*) and fruit bat (*Pteropus giganteus*), were also present, along with the gray langur (*Semnopithecus entellus*), the only primate recorded.

Domestic animals documented included cattle (*Bos taurus*), goats (*Capra aegagrus hircus*), feral dogs (*Canis lupus familiaris*), cats (*Felis catus*), and domestic pigs (*Sus scrofa domesticus*), with grazing activity observed primarily in the peripheral areas. Human activity, divided into villagers, forestry officers, and ecotourists, was recorded within the reserve. Human presence decreased from 28.04% in the first year to 3.92% in the second year of the study.

We observed an increase in the relative abundance of several wildlife species in 2018–2019 compared to 2017–2018, including the Indian peafowl, rufous treepie, jungle babbler, red-vented bulbul, Indian leopard, and small Indian civet. Over the 2 years, 661 images of leopards were captured, identifying 25 individual leopards (8 males and 17 females).

Of the 39 species recorded, 2 were classified as “Vulnerable” (Indian leopard, sambar deer), 1 as “Near Threatened” (striped hyena), and the remaining 36 as “Least Concern” according to the IUCN Red List [36].

The interpolated abundance maps for 2017–2018 showed higher abundance density in the southern and southwestern parts of the study area, whereas in 2018–2019, the abundance density was more evenly distributed across the northern and southern parts (Figure 3a,b).

**Figure 4:**
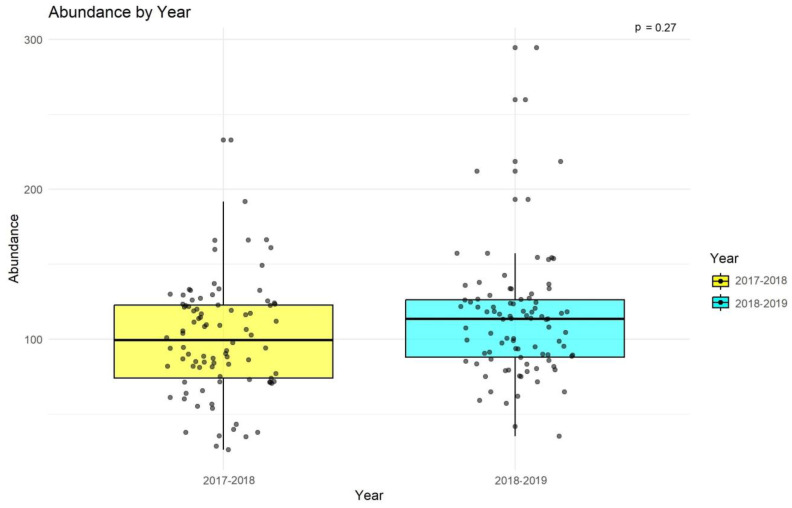
Year-wise statistical comparison of species abundance based on the Kruskal-Wallis test for Jhalana Reserve Forest, Jaipur, India.

There was no significant difference in abundance between the two years (*p* = 0.27; Figure 4).

**Figure 5:**
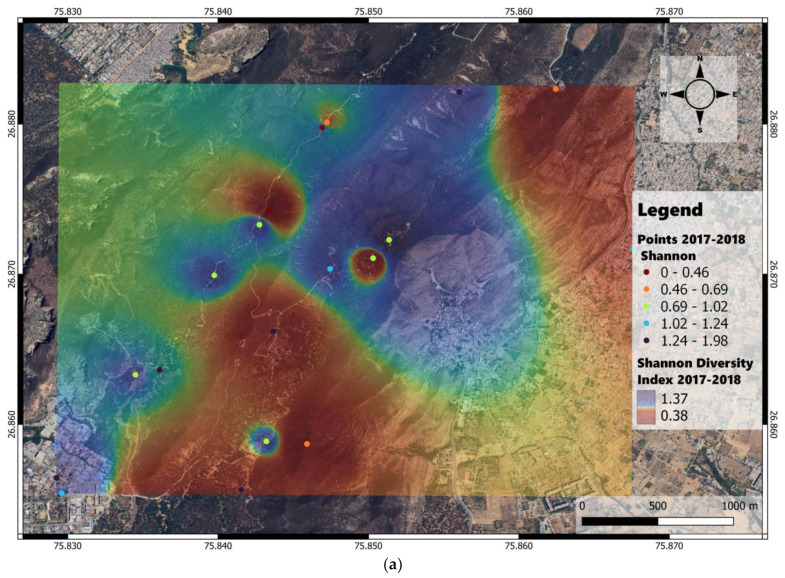

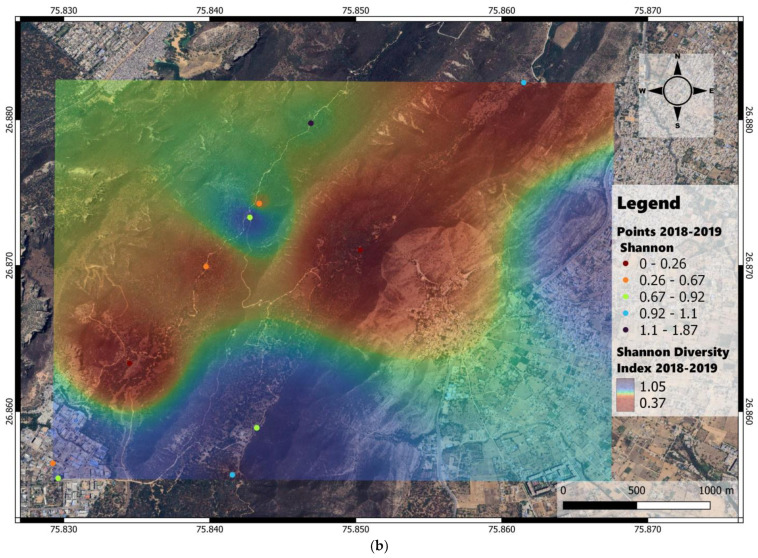
Shannon diversity heat maps derived through inverse distance weighting interpolation applied to recordings of the camera traps deployed at point locations shown on the map of Jhalana Reserve Forest, Jaipur, India. (**a**) Shannon diversity map for 2017–2018 along with the camera trap observation points. (**b**) Shannon diversity map for 2018–2019 along with the camera trap observation points.

The Shannon diversity index maps indicated higher diversity in the northern and central parts of the study area in 2017–2018, while in 2018–2019, diversity was higher in the southern, central, and southeastern parts (Figure 5a,b).

**Figure 6:**
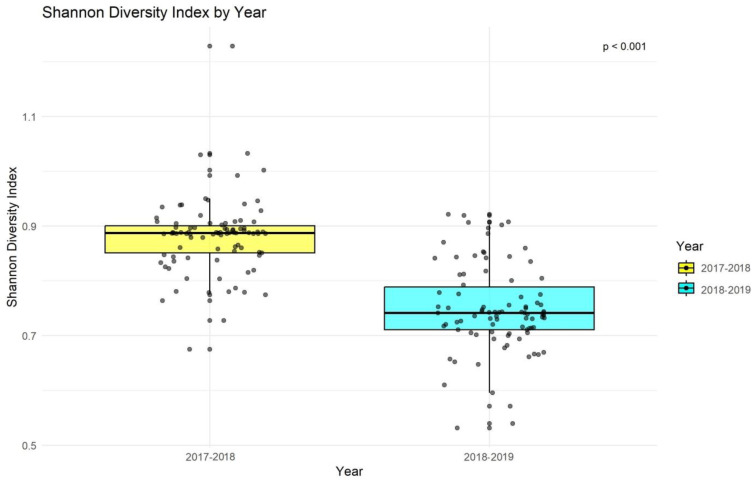
Year-wise statistical comparison for study periods 2017–2018 and 2018–2019 of Shannon diversity index based on the Kruskal-Wallis test for Jhalana Reserve Forest, Jaipur, India.

There was a significant difference in the Shannon diversity index values between the two years (*p* < 0.01; Figure 6). This change in diversity has implications for the prey community of the apex predator, the Indian leopard, and the resource dependence of other species. Importantly, these changes are likely influenced by shifts in waterhole locations, underscoring the role of environmental factors in shaping diversity.

## 4. Discussion

This study is the first to scientifically document the species richness within the JRF, capturing both wildlife and anthropogenic activities to understand the implications of declaring the JRF as India’s first leopard reserve. Despite the semi-arid, human-exploited landscape, the JRF hosts seven carnivore species, although wild herbivores are not naturally present. These findings are consistent with camera trap studies in human-dominated landscapes, such as those in Maharashtra [37].

Without tigers and wolves (*Canis lupus pallipes*), the leopard serves as the apex predator in the JRF. Our results align with those of Karanth and Sunquist [38] and Reddy et al. [39], who noted that leopards thrive where large or medium-sized prey and competitors such as tigers or dholes (*Cuon alpinus*) are absent. Leopards were photographed at all camera traps and distributed throughout the reserve and surrounding areas. Specific observations included a male leopard with an injury in December 2017 who later recovered. Unlike other studies [37,40], we captured leopards’ flanks and facial markings, enabling the individual identification of 17 females and 8 males [22]. We recorded three females with cubs, indicating a resident and reproductive population akin to the findings by Athreya et al. [37].

The diet composition of the JRF leopards shows a dependence on human-associated and domestic animals, such as feral dogs, cats, goats, and cattle [28]. Domestic animals constituted 89% of the leopards’ prey. A survey in Jaipur city reported 36,850 domestic dogs [41], a significant prey source for leopards. Camera trap data corroborate these findings with numerous images of domestic animals within the JRF.

Eighteen villages surround the JRF, and the local community relies on the forest for livelihood, engaging in activities such as grazing and firewood collection, leading to frequent human-wildlife interactions. The Rajasthan Forest Department’s vigilance significantly reduced human activity within the reserve from 28.04% in 2017–2018 to 3.92% in 2018–2019 (Table 1). This resulted in an increased relative abundance of dominant wildlife species in 2018–2019 (Table 1), a promising sign for the future of the JRF. This supports O’Brien and Kinnaird’s [5] assertion that camera traps are effective for monitoring ground-dwelling birds and other wildlife, though they may under-represent arboreal species. We documented more Indian peafowl, jungle babblers, rufous tree-pies, red-vented bulbuls, red-wattled lapwings, and blue-rock pigeons in the second year of the study as compared to the first year (Table 1). However, we are uncertain whether this can be attributed to the erecting of the wall around the JRF or as an artifact of changing environmental conditions and accessibility to the waterholes. This needs to be elucidated in future studies of longer duration.

Striped hyenas, Bengal foxes, and small Indian civets were commonly observed and well-distributed in the JRF. Golden jackals and Indian mongooses were occasionally spotted at waterholes. Hyenas were frequently seen near the southeastern border, close to human habitations. The construction of a 3-m-high wall on the reserve’s border in 2018 by the Rajasthan Forest Department reduced domestic animal intrusions to zero in 2018–2019, though leopards can still traverse the fence. Similarly, human intrusion has reduced considerably, reducing resource competition between the human population and the wildlife of the JRF.

A 2018 survey indicated 90% support for conservation efforts [27]. However, as domestic livestock is a significant food source for carnivores in human-modified habitats [28,42,43], conservation strategies must balance human and wildlife needs. Our results show that integrating wildlife into human-modified landscapes while considering human tolerance is crucial [44]. Research should extend beyond reserve boundaries to include adjacent areas, particularly in the JRF, where urban neighborhoods are contiguous with the forest.

Forest workers are primarily active in the central JRF, involved in road construction, tree planting, water tanker transport to waterholes, and ecotourism route maintenance. Ecotourism, regulated since June 2017 under “Project Leopard”, has contributed to human presence but has managed to minimize wildlife disturbance. Electric vehicles have been introduced to reduce noise impact [31].

Several studies have highlighted the effects of ecotourism on wildlife behavior, including changes in feeding, hormonal responses, habituation, predation, and reproductive success [45,46,47,48]. Further research on the impact of tourism in the JRF will aid in developing responsible tourism policies. It is crucial that we, as stakeholders in the conservation of the JRF, understand and adhere to these policies to ensure the well-being of the wildlife. Continuous monitoring of species richness is vital for tracking protected and endangered species [49,50].

Our study demonstrates that camera trapping is an effective method for estimating and recording the animal diversity of the Jhalana Reserve Forest (JRF). The findings reveal that human-dominated landscapes can sustain and support a consortium of predators. The wildlife in the JRF shares resources with humans outside the reserve forest. Various conservation measures undertaken by the Rajasthan Forest Department have significantly reduced human intrusion. This has led to an increase in the relative abundance of wildlife species in the JRF. Camera traps have proven to be an essential tool for the ongoing monitoring of vulnerable and near-threatened species in the JRF, and this approach can be valuable in other human-shared protected areas to assess the status of wildlife amidst anthropogenic activities. Our results underscore the importance of integrating wildlife into human-modified landscapes while considering human tolerance. It is imperative to study areas adjacent to or on the fringes of protected areas, as there is a continuum between the forests and human habitats. The ongoing monitoring of species richness is vital for tracking protected and endangered species, providing reassurance about the future of the JRF.

## Figures and Tables

**Table 1:** Camera-trapped species in the JRF, along with their photographic records and relative abundance (%) during 2017–2019 (in descending order). Species with (*) are gregarious species.

Common Name	Scientific Name	Family	N 2017_2018	N 2018_2019	Relative Abundance 2017_2018	Relative Abundance 2018_2019
Birds						
Indian Peafowl *	*Pavo cristatus*	Phasianidae	3047	1579	24.62	39.94
Jungle Babblers *	*Turdoides striata*	Leothricidae	9	334	0.07	8.45
Rufous Treepie *	*Dendrocitta vagabunda*	Corvidae	3	310	0.02	7.84
Red-vented Bulbul *	*Pycnonotus cafer*	Pycnonotidae	3	308	0.02	7.79
Red-wattled Lapwing	*Vanellus indicus*	Charadriiidae	1	62	0.01	1.57
Blue Rock Pigeon *	*Columba livia*	Columbidae	3	64	0.02	1.62
Grey Francolin	*Francolinus pondicerianus*	Phasianidae	8	10	0.06	0.25
Cattle Egret	*Bubulcus ibis*	Ardeidae	7	10	0.06	0.25
Eurasian Collared Dove	*Streptopelia decaocto*	Columbidae	0	7	0.00	0.18
Greater Coucal	*Centropus sinensis*	Cuculidae	4	5	0.03	0.13
Shikra	*Accipiter badius*	Accipitridae	2	5	0.02	0.13
White-throated Kingfisher	*Halcyon smymensis*	Alcedinidae	19	2	0.15	0.05
Brahminy Starling *	*Sturnia pagodarum*	Sturnidae	0	1	0.00	0.03
Cinerous Tit	*Parus cinereus*	Paridae	0	1	0.00	0.03
Black Drongo	*Dicrurus macrocercus*	Dicruridae	0	1	0.00	0.03
Jacobin Cuckoo	*Clamator jacobinus*	Cuculidae	0	1	0.00	0.03
Indian Eagle Owl	*Bubo bengalensis*	Strigidae	2	1	0.02	0.03
Long-legged Buzzard	*Buteo rufinus*	Accipitridae	4	0	0.03	0.00
Mammals						
Nilgai *	*Boselaphus tragocamelus*	Bovidae	3377	622	27.29	15.73
Indian Leopard	*Panthera pardus fusca*	Felidae	473	188	3.82	4.76
Small Indian Civet	*Viverricula indica*	Viverridae	10	100	0.08	2.53
Indian Crested Porcupine	*Hystrix indica*	Hystricidae	178	60	1.44	1.52
Bengal Fox	*Vulpes bengalensis*	Canidae	295	48	2.38	1.21
Striped Hyena	*Hyaena hyaena*	Hyaenidae	328	30	2.65	0.76
Black-naped Hare	*Lepus nigricollis*	Leporidae	427	30	3.45	0.76
Golden Jackal	*Canius aureus*	Canidae	0	7	0.00	0.18
Sambar Deer *	*Rusa unicolor*	Cervidae	7	6	0.06	0.15
Indian Mongoose	*Herepestes edwardsii*	Herpestidae	8	5	0.06	0.13
Indian Hedgehog	*Paraechinus micropus*	Erinaceidae	1	1	0.01	0.03
Spotted Deer *	*Axis axis*	Cervidae	157	0	1.27	0.00
Gray Langur	*Semnopithecus*	Pteropodidae	1	0	0.01	0.00
Fruit Bat	*Pteropus giganteus*	Pteropodidae	1	0	0.01	0.00
Domestic Animals						
Cat	*Felis catus*	Felidae	2	0	0.02	0.00
Cattle	*Bos taurus*	Bovidae	449	0	3.63	0.00
Goats	*Capra aegagrus hircus*	Bovidae	48	0	0.39	0.00
Horse	*Equus caballus*	Equidae	18	0	0.15	0.00
Feral Dog	*Canis lupus familiaris*	Canidae	10	0	0.08	0.00
Domestic Pig	*Sus scrofa domesticus*	Suidae	2	0	0.02	0.00
Human						
Humans *	*Homo sapiens*	Hominoidea	3470	155	28.04	3.92
Total			12,374	3953	100	100

## Data Availability

The datasets generated during and/or analysed during the current study are available from the corresponding author on reasonable request.
